# HET0016, a Selective Inhibitor of 20-HETE Synthesis, Decreases Pro-Angiogenic Factors and Inhibits Growth of Triple Negative Breast Cancer in Mice

**DOI:** 10.1371/journal.pone.0116247

**Published:** 2014-12-30

**Authors:** Thaiz Ferraz Borin, Debora A. P. C. Zuccari, Bruna V. Jardim-Perassi, Lívia C. Ferreira, A. S. M. Iskander, Nadimpalli Ravi S. Varma, Adarsh Shankar, Austin M. Guo, Guillermo Scicli, Ali S. Arbab

**Affiliations:** 1 Laboratório de Investigação Molecular no Câncer (LIMC), Faculdade de Medicina de São José do Rio Preto (FAMERP), Avenida Brigadeiro Faria Lima, 5416, São José do Rio Preto, SP, 15090-000, Brazil; 2 Cellular and Molecular Imaging Laboratory, Department of Radiology, Henry Ford Hospital, Detroit, Michigan, 48202, United States of America; 3 Department of Pharmacology, New York Medical College, Valhalla, New York, 10595, United States of America; Southern Illinois University School of Medicine, United States of America

## Abstract

A selective inhibitor of 20-HETE synthesis, HET0016, has been reported to inhibit angiogenesis. 20-HETE has been known as a second mitogenic messenger of angiogenesis inducing growth factors. HET0016 effects were analyzed on MDA-MB-231 derived breast cancer in mouse and *in* *vitro* cell line. MDA-MB-231 tumor cells were implanted in animals’ right flank and randomly assigned to early (1 and 2), starting treatments on day 0, or delayed groups (3 and 4) on day 8 after implantation of tumor. Animals received HET0016 (10 mg/kg) treatment via intraperitoneal injection for 5 days/week for either 3 or 4 weeks. Control group received vehicle treatment. Tumor sizes were measured on days 7, 14, 21, and 28 and the animals were euthanized on day 22 and 29. Proteins were extracted from the whole tumor and from cells treated with 10 µM HET0016 for 4 and 24 hrs. Protein array kits of 20 different cytokines/factors were used. ELISA was performed to observe the HIF-1α and MMP-2 protein expression. Other markers were confirmed by IHC. HET0016 significantly inhibited tumor growth in all treatment groups at all-time points compared to control (p<0.05). Tumor growth was completely inhibited on three of ten animals on early treatment group. Treatment groups showed significantly lower expression of pro-angiogenic factors compared to control at 21 days; however, there was no significant difference in HIF-1α expression after treatments. Similar results were found *in* *vitro* at 24 hrs of HET0016 treatment. After 28 days, significant increase of angiogenin, angiopoietin-1/2, EGF-R and IGF-1 pro-angiogenic factors were found (p<0.05) compared to control, as well as an higher intensity of all factors were found when compared to that of 21 day’s data, suggesting a treatment resistance. HET0016 inhibited tumor growth by reducing expression of different set of pro-angiogenic factors; however, a resistance to treatment seemed to happen after 21 days.

## Introduction

Surgery is the main mode of treatment for breast cancer. Breast conserving surgery is preferred over total mastectomy; however, the decision depends on tumor size, local invasion, lymphnode and distal metastasis [1], [2]. Because of the hypervascular nature of breast cancer, especially metastatic breast cancer (MBC) and associated active angiogenesis, antiangiogenic treatment has been added as an adjuvant to control angiogenesis, and to inhibit tumor growth [3]. Different targets have been selected to control abnormal angiogenesis [3], [4]. Regrettably, benefits of antiangiogenic therapy are at best transitory, and this period of clinical benefit (measured in weeks or months) is followed by restoration of tumor growth and progression. Studies suggest that inhibition of angiogenesis is even a driving force for tumor conversion to a greater malignancy, reflected in heightened invasion and dissemination into surrounding tissues and in some cases increased lymphatic and metastatic activities [5]. Therefore, any agent that inhibits multiple angiogenesis pathways and has also anti-tumoral activity might be helpful in controlling malignant tumors.

N-hydroxy-N′-(4-butyl-2 methylphenyl) formamidine (HET0016) has been reported to be a highly selective inhibitor of 20-hydroxy arachidonic acid (20-HETE) synthesis that involves enzymes of the cytochrome P450, family 4, subfamily A (CYP4A) and CYP4F families [6]. HET0016 was found to inhibit angiogenic responses to several growth factors as well as angiogenesis in the cornea induced by implanted human U251 glioma cells and gliosarcoma [7], [8]. They also studied whether the CYP4A-20-HETE system can contribute to the regulation of endothelial progenitor cells (EPCs). Stem cells isolated from human umbilical cord that were CD133+/CD34+ and thus considered EPCs [9]. These cells contained both immunoreactive CYP4A11 and CYP4F2, and secreted 20-HETE. On the other hand, exogenous 20-HETE was shown to increase proliferation and migration of EPCs, and enhanced tube formation in an *in* *vitro* matrigel model of angiogenesis [10]. This in-vitro angiogenesis was abolished by HET0016 and by other 20-HETE competitive antagonist, suggesting that 20-HETE is involved in EPC-induced angiogenesis. EPC’s proliferation and migration induced by vascular endothelial growth factor (VEGF) and migration induced by stromal cell-derived factor 1 alpha (SDF-1α) were also abolished by HET0016.

Reports on effects of HET0016 in breast cancer do not exist currently in the literature. However, reports indicate that the agent is also effective against other cancers such as non-small cell lung carcinoma and renal adenocarcinoma [11], [12]. Recent studies showed the presence of cytochrome P450 isoforms essential for the synthesis of 20-HETE in different breast cancer cell lines [13]–[15], therefore, it is expected that HET0016 will be effective against breast cancer. Thus, HET0016 appears to have potential as a novel adjuvant therapeutic agent for breast cancer.

The purposes of this study were to determine the effect of HET0016 in an *in* *vivo* model of triple negative breast cancer developed by MDA-MB-231 in nude mouse, and the expression of different pro-angiogenic factors/cytokines in mammary tumor and *in* *vitro* system.

## Materials and Methods

All experimental procedures were approved by the Institutional Animal Care and Use Committee and Institutional Review Board of Henry Ford Hospital (IACUC No. 1228). All efforts were made to ameliorate suffering of animals.

### Cell Line

Human breast cancer MDA-MB-231 cell line was originally obtained from the American Type Tissue Culture Collection (ATCC, Manassas, VA, USA). These cells line were grown in Dulbecco’s modified eagles medium (DMEM) high glucose (4.5 g/L) (GIBCO, Grand Island, NY, USA), supplemented with 10% fetal bovine serum (FBS) (GIBCO, Grand Island, NY, USA), 2 mM glutamine (GIBCO, Grand Island, NY, USA) and 100 U/mL penicillin and streptomycin (GIBCO, Grand Island, NY, USA) at 5% CO_2_ at 37°C in a humidified incubator. For implantation the cells were harvested and re-suspended in Roswell Park Memorial Institute (RPMI) (GIBCO, Grand Island, NY, USA) serum free media. A total of three million cells in 100 µL were implanted into the animal’s right flank.

For *in* *vitro* study ten million cells were plated in 75 cm^2^ flask containing 10 mL of RPMI serum free media containing 10 µM of HET0016 for 4 and 24 hrs. The same assay was performed for controls without HET0016 treatment, and then the cells and their media were collected, frozen and stored at −80°C. Cellular viability was also determined in cancerous vs non-cancerous breast cells. Hundred thousand triple negative breast cancer (MDA-MB-231) and non-tumorigenic breast (MCF-10A) cells per mL were subjected to different doses of HET0016 (5 µM and 100 µM) for four hours in serum free media. Cell viability was determined by trypan blue dye exclusion tests. The experiments were repeated by two independent investigators on two different dates (**S1 Fig**).

### Animal Model and Treatment schedules

Athymic nude female mice (n = 28) 6–8 week-old and weighing 20–25 grams obtained from Charles River laboratory (Frederick, MD) were used in all experiments. After tumor implantation the animals received treatment with HET0016 (10 mg/kg per day) or phosphate buffered saline (PBS) containing 10% of dimethyl sulfoxide(DMSO) (Sigma, St. Louise, MO, USA) and 10% cremophor (Sigma, St. Louise, MO, USA) as a vehicle control intraperitoneally (IP) for five days during the week (Monday through Friday). To better evaluate the effect of treatment, the animals were divided into four treatment groups receiving early treatment (beginning on the day of tumor inoculation) or delayed (on day 8 after inoculation of the tumor) and maintained for 21 or 28 days. Group 1 (n = 6) starting treatment early on day 0 until day 21; Group 2 (n = 4) also starting treatment early on day 0, however, was maintained for 28 days; Group 3 (n = 6) starting treatment delayed, after tumor establishment on day 8 until day 21 and Group 4 (n = 4) also starting treatment delayed on day 8, however, was maintained for 28 days. Eight animals were used as control receiving vehicle, four were maintained for 21 days and others four were maintained for 28 days.

### Body Weight and Tumor Growth

Body weight (an indicator of overall animal health) was measured twice weekly. An electronic digital scale (Thermo Fisher Scientific, Rockford, IL, USA) was used to weigh mice, and electronic digital calipers were used to measure the tumor size. The tumor masses were measured at day 7, 14, 21 and 28. Tumor volume was calculated using the formula volume  =  (length × width)/2. Tumor growth curves and growth rate were calculated for each group.

### Single Photon Emission Computed Tomography (SPECT) Study

Succinimidyl 6-hydrazinopyridine-3-carboxylate hydrochloride was synthesized and conjugated with rat VEGF-c, a recombinant rat VEGF-c (Prospec, Rehovot, Israel), lacking the N- and C-terminal extensions containing only the middle VEGF homology domain, forms primarily non-covalently linked dimers. This protein is a ligand for both VEGF receptor 2 or kinase insert domain receptor (VEGFR-2/KDR) and VEGF receptor 3 or Fms-related tyrosine kinase 4 (VEGFR-3/FLT-4). The protein conjugate was purified with a Centricon C-3 diafilter with a 3,000 molecular weight cutoff. Then, the nicotinyl hydrazine conjugate of VEGF was radio labeled with Tc-99m-pertechnetate in the presence of tricine and stannous chloride as reported by Blankenberg et al. [16]. Animals were anesthetized using ketamine/xylazine (100/10 mg/kg) and received 0.5 mCi of Tc-99m-HYNIC-VEGF-c through tail vein injection in a volume of 100 µL. One hour after injection, SPECT images were obtained using a modified PRISM 3000 gamma camera dedicated to animal studies and fitted with multi pinhole collimators (Bioscan, Washington DC, USA). The following image parameters were used: 256×256 matrix, 360° rotations, 180 sec per projection and 4×9 cm FOV. The total time required for acquiring SPECT was about 10 minutes. After the SPECT analysis animals were euthanatized and tumors were collected for further analysis. The projection images were reconstructed with HiSPECT software (Bioscan, Washington DC, USA).

### Image Analysis

Multiplanar reconstruction was performed using a slice thickness of 0.8 mm. Volumetric images were created by adding slices from the entire animal, dorsal to ventral. The region of interest was drawn on the right flank (site of tumor) and on contralateral intact flank and the radioactivity was measured. Radioactivity in tumor areas was normalized to corresponding contralateral region to produce a ratio of radioactivity in tumor to normal tissues and then the ratio was normalized to corresponding activity ratio in control tumors. The activity in the tumor was expressed in percent of activity in control tumor.

### Histopathology and Immunohistochemistry

For histology and immunohistochemistry, following euthanasia animals were perfused with PBS and then 3% paraformaldehyde (Acros Organics, New Jersey, USA), tumor was collected, and fixed in 3% paraformaldehyde containing 3% sucrose (Fisher Chemical, Waltham, MA, USA). Tissues collected from radioactive animals were kept in a shielded area for decay, and then tissue sections were prepared for paraffin blocks and sectioning. Standard immunohistochemical staining procedures were performed as recommended by the suppliers of primary antibodies. The following antibodies were used to delineate the expression of corresponding antigens: Von Willebrand factor (vWF) (Dako, Carpinteria, CA, USA) and Ki67 (Millipore, Billerica, MA, USA).

### Tumor micro-vessel density (MVD) and proliferative cell analysis

Micro-vessels were detected by immunohistochemical staining with vWF. Each cell positive for vWF was considered to be a micro vessel. Five “hot spots” [area with highest vessel concentration] from each slide were identified, and vWF positive areas were counted by two independent observers. The total histological area was noted, and MVD was calculated as previously described [17].

Tumors were categorized in relation to cellular proliferation according to the staining of Ki-67. All Ki-67 positive cells were counted from each photographed area. The number of Ki-67 positive cells was normalized to the area of photomicrography.

### Protein Extraction

Protein extracts were harvested from MDA-MB-231 cells, cells culture media (supernatant) and tumors from the animals. The cells stored at −80°C after treatment were thawed, washed twice in cold PBS and lysed using NP40 lysis buffer (Invitrogen, Grand Island, NY) plus protease inhibitor cocktail (Sigma, St. Louise, MO, USA) according to the manufacturer’s instructions. The cell lysates were centrifuged for 10 min at 14,000 g at 4°C. The supernatant was preserved and the protein concentration was determined. Ultrafiltration devices (Sartorius Stedim Biotech, Goettingen, Germany) were used to concentrate proteins. Proteins greater than 3 kDa were retained by ultrafiltration membrane, then concentration was determined.

For protein extraction from tumor tissues, selected animals were perfused only with ice cold PBS following euthanasia and half of the tumor was snapped frozen and kept in −80°C. Another half of the tumor was fixed in 3% paraformaldehyde for histopathology. Total proteins were extracted from the tumor using scrapping manual over dry ice and 2x RayBio cell-lysis buffer plus protease inhibitor cocktail (Roche) according to the manufacturer’s instructions (RayBiotech, Norcross, GA, USA).

Total protein concentration for all samples (cells, media and tumor) was measured using BioRad Protein Assay (Bio-Rad Laboratories, Hercules, CA, USA) using Bovine Serum Albumin (BSA) as a standard.

### Protein analysis by Human cytokine antibody array

Human cytokine antibody array kit from RayBiotech, Inc (RayBiotech, Norcross, GA, USA) was used for analysis the expression profiles of the proteins extracted from the tumor tissue, cell lysate and from media in cell culture. The membrane-based quantitative antibody array has a standard membrane spotted with 20 cytokine capture antibodies, and the detection is based on chemiluminescence labeling. 250 µg of protein sample was applied to the membrane according to the manufacturer’s instructions, and the chemiluminescent reaction was detected using Multispectral Imaging System (Kodak Carestream, MA, USA). Images were analyzed using Image J software (NIH), and the density of each signal was normalized with the positive controls on the membrane as well as to the corresponding values in the control animals. The list of the 20 factors that were used to evaluate the angiogenic process during HET0016 treatment and their respective results are shown in **Table 1**.

**Table 1:** Human cytokine antibody array kit (RayBiotech, USA) contending 20 different growth factors/cytokines were used for analysis of protein expression profile in tumor tissue and cells lysate.

Pro-angiogenic Factors	Tumor 21 days Treatment		Tumor 28 days Treatment		MDA-MB-231 cells			
	EARLY (0–21 d)	DELAYED (8–21 d)	EARLY (0–28 d)	DELAYED (8–28 d)	4 hrs		24 hrs	
					Cell lysed	Super natant	Cell lysed	Super natant
**Angiogenin**	Ns	↓ p = 0.0314	ns	↑ p = 0.0200	↓ p = 0.0378	ns	↓ p = 0.0020	ns
**Angiostatin**	↓ p = 0.0186	↓ p = 0.0006	ns	↑ p = 0.0020	ns	ns	↓ p = 0.0301	ns
**Angiopoietin-1**	↓ p = 0.0315	↓ p = 0.0043	ns	↑ p = 0.0004	ns	ns	ns	ns
**Angiopoietin-2**	Ns	↓ p = 0.0313	ns	↑ p = 0.0018	ns	ns	↓ p = 0.0045	ns
**G-CSF**	↓ p = 0.0127	↓ p = 0.0075	ns ↓ p = 0.0991	ns	ns	ns	ns	ns
**PDGF-AA**	↓ p = 0.0370	↓ p = 0.0114	Ns	ns	ns	ns	ns	ns
**PDGF-RA**	ns	↓ p = 0.0306	Ns	ns	ns	*	ns	*
**RANTES**	ns	↓ p = 0.0303	Ns	ns	ns	*	↓ p = 0.0050	*
**bFGF**	ns	↓ p = 0.0248	Ns	ns	ns	ns	↓ p = 0.0086	ns
**EGF**	↓ p = 0.0321	↓ p = 0.0019	Ns	ns	ns	ns	↓ p = 0.0184	ns
**EFG-R**	↓ p = 0.0338	↓ p = 0.0012	↑ p = 0.0004	↑ p = 0.0154	ns	*	ns	*
**IGF-1**	ns	ns	↑ p = 0.0125	↑ p = 0.0134	ns	ns	↓ p = 0.0228	ns
**MMP-9**	↓ p = 0.0046	↓ p<0.0001	ns ↓ p = 0.0589	ns	ns	ns	ns	ns
**SDF-1α**	↓ p = 0.0277	↓ p = 0.0089	ns	ns	ns	ns	ns	ns
**Tie-1**	↓ p = 0.0232	↓ p = 0.0007	ns	ns	ns	*	ns	*
**Tie-2**	ns	↓ p = 0.0089	ns	ns	ns	*	ns	*
**VEGF-A**	ns	↓ p = 0.0164	ns	ns	ns	ns	↓ p = 0.0416	ns
**VEGF-C**	ns	↓ p = 0.0233	ns	ns	ns	ns	↓ p = 0.0152	ns
**VEGF-R2**	ns	ns	ns	ns	ns	*	ns	*
**VEGF-R3**	↓ p = 0.0016	↓ p = 0.0021	ns	ns	ns	*	↓ p = 0.0166	*

G-CSF: colony stimulating factor; PDGF-RA: platelet-derived growth factor receptor, alpha polypeptide; PDGF-AA: platelet-derived growth factor alpha polypeptide; bFGF: fibroblast growth factor (basic); EGF: epidermal growth factor; EGF-R: epidermal growth factor receptor; IGF-I: insulin-like growth factor 1; MMP-9: matrix metallopeptidase 9; SDF-1α: Stromal cell-derived factor 1 alpha; Tie-1: receptor tyrosine kinase of angiopoietin-2; Tie-2: receptor tyrosine kinase of angiopoietin-1; VEGF-A: vascular endothelial growth factor A; VEGF-C: vascular endothelial growth factor C; VEGF-R2: vascular endothelial growth factor receptor 2; VEGF-R3: vascular endothelial growth factor receptor 3. All antibodies are prepared in duplicate. ns: no significant; *not evaluated.

### Statistical Analysis

Comparison between drug and vehicle treated groups was done by using one way ANOVA with PLSD Post hoc test. All data are expressed as mean ± standard error of mean (SEM). Any p-value of ≤0.05 was considered significant.

## Results

### Treatment effect of HET0016 on tumor growth

**Figure 1:**
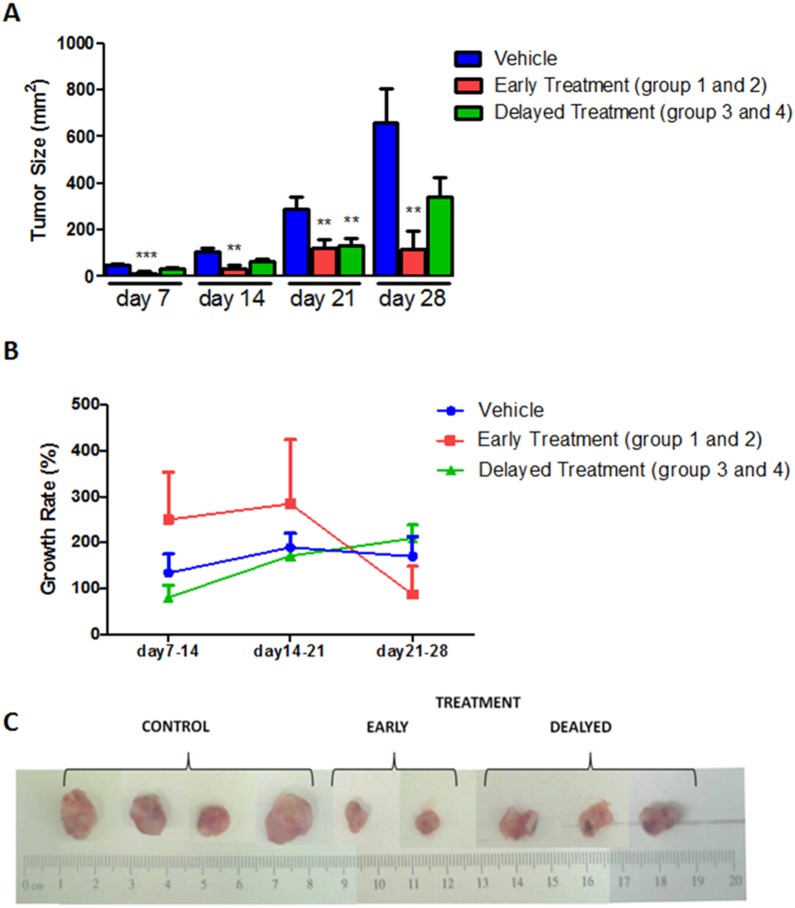
HET0016 treatment reduced tumor growth in breast cancer nude mice. **A)** HET0016 treatment decreased tumor volume in both the early and delayed treatment groups. Significant difference was observed between control and treatment groups as early as 7 days of treatment. When compared the tumor growth rates (**B**), animal that received delayed treatment showed rapid growth at late stage of treatment. **C)** Representative samples of mammary tumors developed by MDA-MB-231 cells implantation on the right flank of mice. **p<0.001, ***p<0.0001.

HET0016 treatment decreased tumor volume in all animals in both early and delayed treatment groups (**Fig. 1A and 1C**). Significant difference was observed between control and treatment groups as early as 7 days of treatment (p = 0.0012). Early treatment groups (groups 1 and 2) showed this difference during all the time points (p≤0.01). Three of ten animals of early treatment groups showed no tumor at the end of the study, although small tumors were detected on day 7 and the tumor disappeared between day 14 and 21. On the other hand, tumors were significantly larger in the delayed treatment groups (groups 3 and 4) compared to that of corresponding tumors in early treatment groups (Day 21, p = 0.015). This indicates possible development of resistance after day 21 of treatment. When compared the tumor growth rates (**Fig. 1B**), animals that received delayed treatment showed the similar growth rate to that of control group. On the other hand, early treatment group showed decline of tumor growth rate at later stage of treatment. However, no significant differences in the growth rates were observed (p>0.05). In vitro study also indicated toxic effect of HET0016 only in cancerous cells S1 **Fig** **.**

### SPECT data

**Figure 2:**
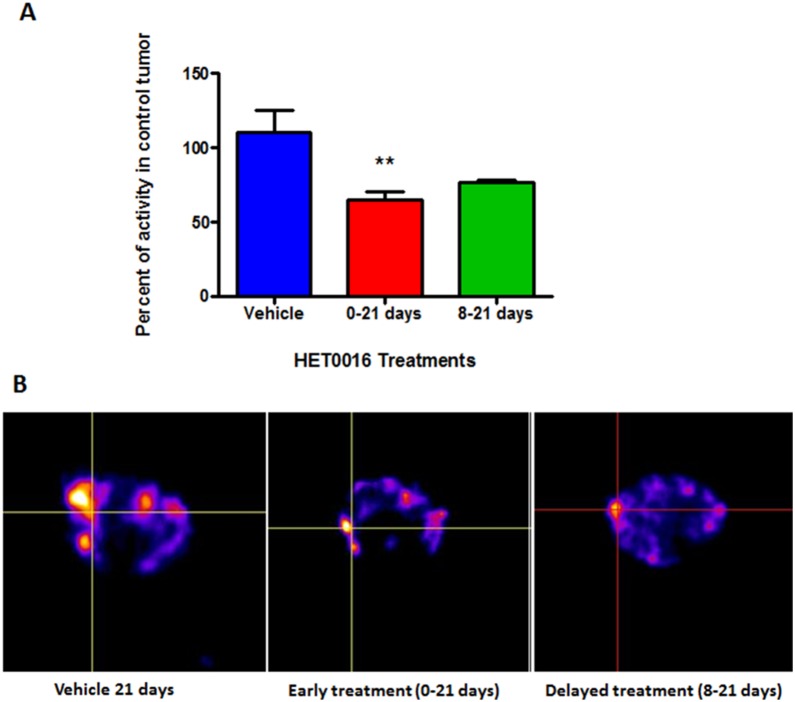
SPECT analysis of *in* *vivo* accumulation of Tc-99m-HYNIC-VEGF-c. One hour after injection, SPECT images were obtained using dedicated animal scanner. **A)** Semi-quantitative analysis of total radioactivity in the tumors normalized to corresponding control tumors showing the intensity of radioactivity in the vehicle and HET0016 treated groups. **B)** Images of vehicle treated mice showed increased accumulation of Tc-99m-HYNIC-VEGF-c compared to that HET0016 treatment groups (early and delayed). Intersection of lines indicates the tumor. Error bars: ± standard error, **p = 0.0131.

*In vivo* SPECT scan using Tc-99m tagged with VEGF-c was performed in randomized animals on early and delayed treatment groups of 21 days to determine whether there were any changes in the expression of VEGFRs. The bio distribution of tumor radioactivity was normalized to contralateral muscles and corresponding ratio in control tumors showed significant lower radioactivity (65.1±5.2%, p = 0.0131, n = 6) at early treatment compared with control 100±20.9% (n = 4) (**Fig. 2A and B**). The intensity of radioactivity on delayed treatment was 76.4±1.8% (n = 2), but statistically significant difference was not achieved (p>0.05).

### Tumor micro-vessel density (MVD) and proliferation cell

**Figure 3:**
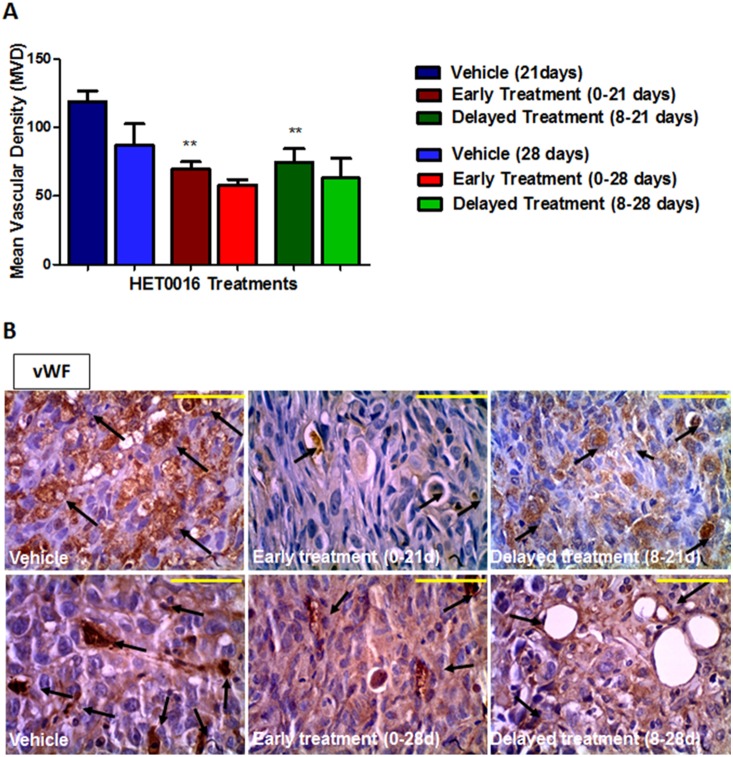
Immunohistochemistry stained with vWF in vehicle and HET0016 treated tumors groups. **A)** Quantitative estimation of Mean Vascular Density. **B)** Top images were taken from tumor of 21 days and bottom images were taken from tumor of 28 days. The arrows shows micro-vessels. Error bars: ± standard error, **p<0.001. Scale bar = 100 µm.

To evaluate the tumor neovascularization, MVD was quantified by using vWF immunohistochemistry following 21 and 28 days of HET0016 treatment. The analysis performed by 21 days showed around 35% lower vWF positive areas in both treatment groups (1 and 3) compared to the vehicle treatment (p<0.005) (**Fig. 3A**). However, significant difference was not achieved when compared the treatment groups from 28 days (2 and 4) (**Fig. 3B**).

**Figure 4:**
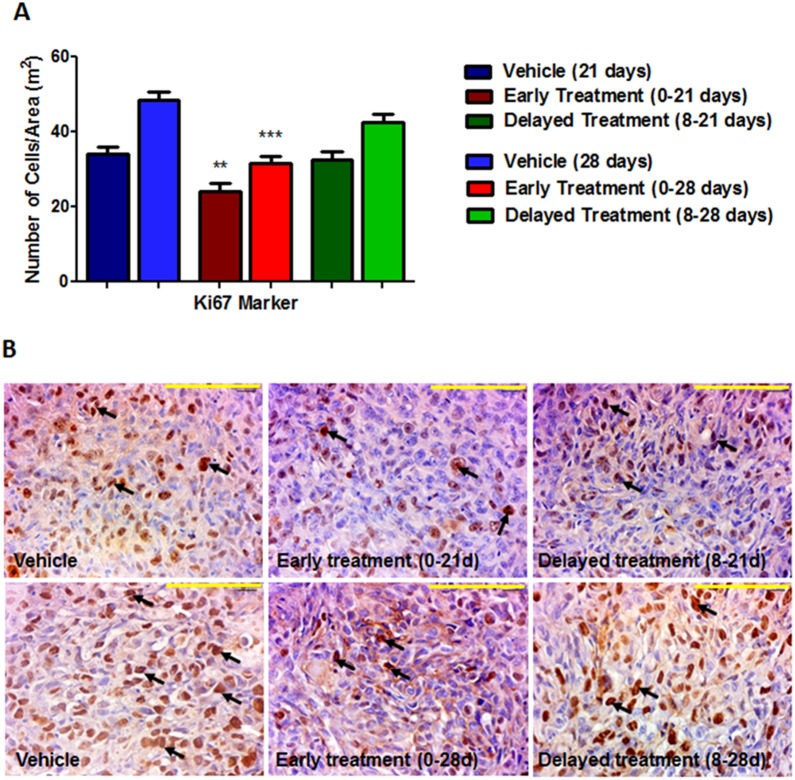
Immunohistochemistry stained with Ki-67 in vehicle and HET0016 treated tumors groups. **A)** Quantitative estimation of cell proliferation showing number of cells per area (m^2^). **B)** Top images were taken from tumor of 21 days and bottom images were taken from tumor of 28 days. The arrows shows cellular nucleus stained. Error bars: ± standard error, **p<0.001. Scale bar = 100 µm.

Tumor cell showed lower proliferation (Ki-67 labelling cells) on early treatment groups (group 1, p = 0.0022 and group 2, p<0.0001) when compared with the control group. (**Fig. 4**). However, the delayed treatment groups (3 and 4) did not show any difference compared to control group.

### Pro-angiogenic growth factors / Protein array

**Figure 5:**
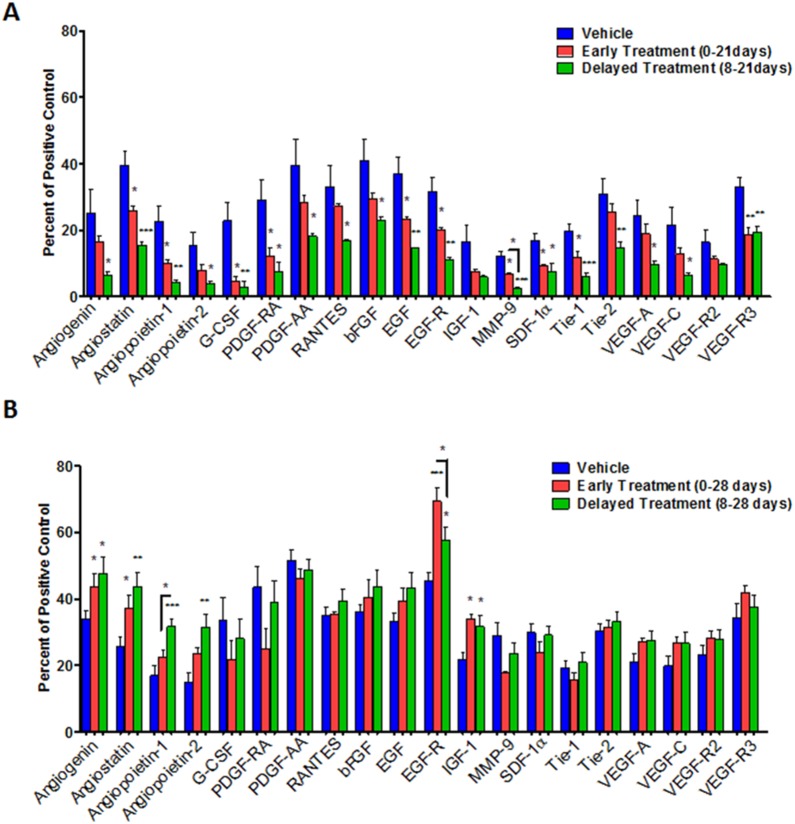
Quantitative estimation of growth factors/cytokines in protein extracted from (A) 21 and (B) 28 days of breast tumor using a custom designed membrane based protein array. Error bars: ± standard error, *p<0.05, **p<0.001, ***p<0.0001.

**Figure 6:**
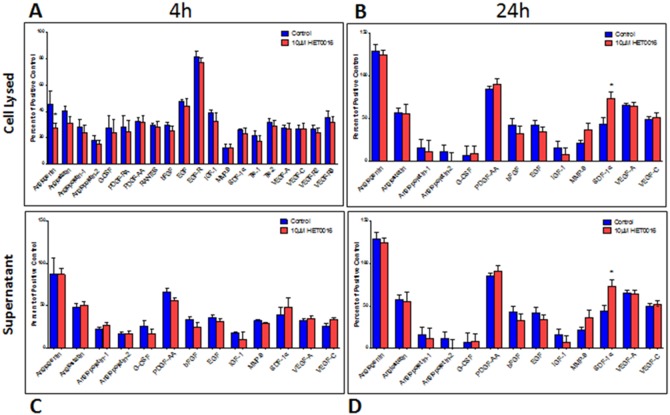
Quantitative estimation of growth factors in protein from cell lysate and supernatant using a custom designed membrane based protein array kit. Cell lysed (**A** and **B**); supernatant (**C** and **D**). Collected after 4 h of treatment 10 µm of HET0016 (**A** and **C**) and 24 h (**B** and **D**). Error bars: ± standard error. *p<0.05.

Angiogenesis related growth factors (**Table 1**) were estimated in the tumors using a membrane based protein array kit. Tumors were collected on different days after treatments to determine whether there are any differences in the expression of factors related to angiogenesis and whether there is any indication of tumor resistance to long term treatments. **Fig. 5A** shows the comparison of expressed proteins among control, early and delayed treated tumors those are collected on day 21. **Fig. 5B** shows the comparison of expressed proteins among control, early and delayed treated tumors those are collected on day 28. Expression of these proteins was also determined in MDA-MB-231 cells line treated with HET0016 for 4 and 24 hours (**Fig. 6**). Overall, with 21 days of treatment almost all factors evaluated related to angiogenesis in the both treated groups showed significantly lower expression compared to control (**Fig. 5A**), including angiostatin, angiopoietin-1, granulocyte-colony stimulating factor(G-CSF), platelet-derived growth factor chain AA (PDGF-AA), epidermal growth factor (EGF), EGF receptor (EGF-R), matrix metallopeptidase 9 (MMP-9), SDF-1α, receptor tyrosine kinase with immunoglobulin-like and EGF-like domains 1 (Tie-1), and VEGF-R3. In addition, angiogenin, angiopoietin-2, platelet-derived growth factor receptor alpha (PDGF-RA), regulated on activation of normal T cell expressed and secreted (RANTES), basic fibroblast growth factor (bFGF), receptor tyrosine kinase with immunoglobulin-like and EGF-like domains 2 (Tie-2), VEGF-A, VEGF-C were found decreased only on delayed treatment group (p<0.05) compared to control group. MMP-9 also showed significant difference (p = 0.0261) when compared between early and delayed treatment groups at 21 days. On the other hand, tumors treated for 28 days did not show decreased expression of factors related to angiogenesis in treated groups (**Fig. 5B**). Rather a few factors such as angiogenin, angiostatin, angiopoietin-1 and -2, EGF-R and insulin-like growth factor 1 (IGF-1) showed significantly (p≤0.03) increased expression in treated tumors compared to that of control groups. PDGF-RA and MMP-9 were decreased on early treatment group but statistically significant difference was not achieved (p = 0.0991 and p = 0.0589, respectively). Nonetheless, angiopoietin-1 and EGF-R had significance difference (p = 0.04) between early and delayed treatment groups at 28 days.

Protein expression determined from cell lysate after treating them with HET0016 for 4 hrs showed significant (p = 0.0378) decreased in the level of angiogenin only compared to that of control untreated cells, however, collected supernatant did not show any changes between treated and untreated groups (**Fig. 6A and 6C**). On the other hand, when the cells were treated with HET0016 for 24 hrs, expression of almost all factors was significantly decreased compared to that of untreated cells, however, corresponding supernatant showed increased level of SDF-1α only (**Fig. 6B and 6D**).

The similar expression profile was observed between 24 hrs in cell lysate and in tumors following 21 days of treatment, where angiogenin, angiostatin, angiopoietin-2, RANTES, bFGF, EGF, IGF-1, VEGF-A, VEGF-C and VEGF-R3 showed significant difference (p≤0.05). All those data are summarized in **Table 1**.

**Figure 7:**
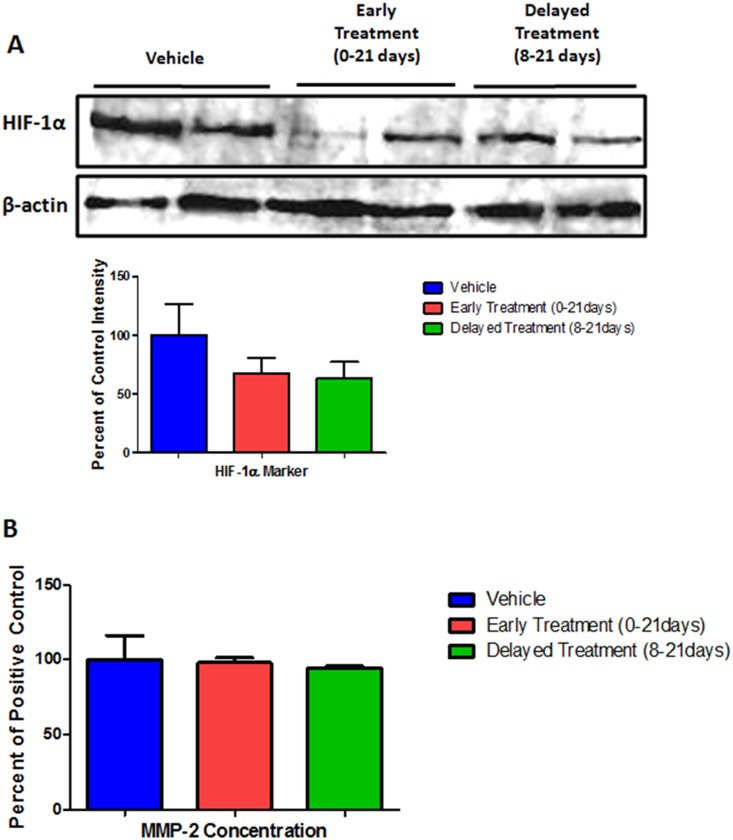
Determination of (A) HIF-1 and (B) MMP-2. No significant differences were observed among the treatment groups.

ELISA and Western blot assays were also performed to determine the hypoxia inducible factor-1 alpha (HIF-1α) and MMP-2 protein expression, but did not show any significant difference (**Fig. 7A and B**). These proteins were not included in the membrane analysis due to cross reactivity.

## Discussion

In the present study, we demonstrated that HET0016 treatment decreased tumor growth and cell proliferation in breast cancer xenograft model. HET0016 has been reported to be a selective competitive inhibitor of the synthesis of 20-HETE [18], [19]. 20-HETE, a metabolite of arachidonic acid (AA) produced by the CYP4A and CYP4F enzyme families, may act as a second messenger for the vasoactive and mitogenic responses of some growth factors, including angiotensin II, norepinephrine, endothelin, vasopressin, serotonin, EGF, and be an important mediator of VEGF, inducing angiogenesis [7], [20], [21]. Our results corroborate with Guo et al. [8] in which chronic administration *in* *vivo* of HET0016 (10 mg/kg/day) reduced the volume of 9L gliosarcoma, accompanied by mitotic and vascularization reduction, increase in the tumor apoptotic index.

In addition, our results showed significant lower VEGF-R2/3 expression by SPECT imaging *in* *vivo* using Tc-99m-HYNIC-VEGF-c. Cancer cells are known for their ability to produce autocrine growth factors that contribute to their abnormal growth rate and 20-HETE in a dose-dependent manner induced endothelial cells (ECs) proliferation, increasing VEGF expression and its release activated by VEGF-VEGF-R2 pathway [20]. VEGF-C expression is closely related to lymphangiogenesis and lymphatic metastasis in a variety of human tumors, binding to VEGF-R2 or VEGF-R3 to induce angiogenesis [22]. Furthermore, VEGF-C overexpressed was observed in breast cancer specimens compared to adjacent normal mammary glands correlating significantly with lymphatic vessel invasion and survival rate [23]. Recent studies suggest that both receptors can contribute to tumor progression and has been shown expressed not only in vascular EC but also in cancer cells [23], [24]. In response to their specific ligands, VEGF-R2 can modulate vascular endothelial survival, proliferation, migration and the formation of vascular tubes [25].

Smith et al. [26] had showed that angiogenic sprout in solid tumors, particularly in breast carcinomas, is regulated not only by VEGF-R2, but also by VEGF-R3. These pre-clinical assays have shown that the overexpression of VEGF-R2 and VEGF-R3 is found in both blood and lymphatic conduits, which imply that the mechanism of action of VEGF signaling inhibitors probably occurs more importantly in tumor vessels rather than tumor cells. Additionally, the up-regulation of VEGF-R3 observed in cancer blood vessels point out the possibility to add an anti-angiogenic therapy that can target both VEGF-R2/VEGF-R3. Decreased expression of VEGF-R2 and VEGF-R3 during HET0016 treatments in both early and delayed period of treatment found in our results might indicate effectiveness of HET0016 in controlling both vascular and lymph angiogenesis.

Several studies propose that 20-HETE, contributes directly and indirectly to angiogenesis in breast cancer by effective growth inhibition in EC [7], [19], [20], [27]–[29]. Our results showed that HET0016 treatment reduced tumor MVD when compared to that of vehicle treatment group. The MVD quantification is associated with an unfavorable survival prognosis in breast cancer and it has been correlated with new vessel formation and the occurrence of metastasis [24].

Angiogenesis plays a key role in the initiation and progression of tumors, and metastasis. All of these characteristics require interaction with its microenvironment controlled by different cytokines and growth factors, such as HIF-1α, VEGF, SDF-1, placental growth factor (PIGF), and PDGF, angiopoietins, FGFs and transforming growth factor- β (TGF-β) [28]. These processes depends upon a balance of angiogenic stimulators and inhibitors, thereby we studied 20 factors related to angiogenesis to evaluate the behavior of them following 21 and 28 days of treatment with HET0016 in breast cancer in *in* *vivo* model. We also studied treatment from the *in* *vitro* study, in earlier stage. Growth factors as G-CSF, PDGF-AA/PDGF-RA, bFGF, EGF/EGF-R, IGF-1, SDF-1α, VEGF-A/VEGF-R2, VEGF-C/VEGF-R3, angiogenin, angiopoietin-1/Tie-2, RANTES, MMP-9 were considering as pro-angiogenic factors and angiostatin, angiopoietin-2/Tie-1 were considered as inhibitors of the angiogenic process. Lower expression of most of the stimulating factors was observed in 21 days compared to control (also similar results were found *in* *vitro* study). However, after 28 days the pro-angiogenic factors showed almost no difference compared to control, suggesting a treatment resistance.

Studies have shown that HET0016 may inhibit angiogenic factors such as VEGF, EGF, FGF-2 [7], [27]. Given the complexity of the angiogenic process, we looked for to evaluate several pro-angiogenic factors to identify how their behaviors are during the treatment. However, current consensus is that VEGF is the most important pro-angiogenic factor, and it can be modulated and stimulated by several other growth factors. VEGF expression by tumors is up regulated by hypoxia and is often elevated near the areas of tumor necrosis. Hypoxia activates a HIF-1α binding sequence in the VEGF promoter, which leads to VEGF mRNA transcription and stability. Guo et al. [27] showed that 20-HETE first caused increase in VEGF, which in-turn causes the up-regulation of HIF-1α. The resulting activation of HIF-1 in these cancers leads to the transcriptional induction, not only of VEGF and VEGFRs, but also endothelin-1, angiopoietins, and angiopoietin receptors (Tie-1 and -2).

Like VEGF, angiopoietin-1 is an endothelial cell specific growth factor, in which induces EC to recruit pericytes and smooth muscle cells to become incorporated in the vessel wall by producing PDGF and other factors when Tie-2 is activated by angiopoietin-1 [30]. Blockade of the stabilizing action of angiopoietin-1 may contribute to tumor vessel regression. Blockade of angiopoietin-1 action by its antagonist angiopoitein-2 also may contribute to tumor vessel regression [31]. However, in spite of our findings which showed a substantial reduction in the tumor size (especially in early treatment groups) and number of its vessels, the expression of both angiopoietin were increased between 21 to 28 days of treatment (mostly prominent in delayed treatment group).

HET0016 was effective as a treatment for 21 days to completely inhibit the growth of 30% of the treated tumors and to decrease a set of pro-angiogenic proteins, especially, EGF/EGF-R. Chronic inhibition of 20-HETE signaling decreased EGF-R phosphorylation in mouse cystic kidneys suggesting the 20-HETE coupling and transactivation of EGF-R [32]. Similar effects were also found in U251 glioma cells, where 20-HETE inhibition by HET0016 reduced an EGF-R activation and, subsequently proliferation stimulated by EGF [27], [33]. On the other hand, preceding studies have indicated that exacerbated signaling of multiple members of the EGF-R family often causes uncontrolled proliferation of cancer cells, which might justify the reason why the tumor became resistant at 28 days, when the levels of EGF/EGF-R were higher [12].

Another important factor, angiostatin, may have contributed for tumor reduction as well. Angiostatin, an internal fragment of plasminogen inhibits EC proliferation and metastatic tumor cell growth [34]. It is generated through proteolytic cleavage of circulating plasminogen by a series of enzymes from the tumor environment, including the MMPssuch as MMP-2, MMP-7, MMP-9, and MMP-12 [35]. Zhang et al. [36] in their studies showed that angiostatin increase was associated with inhibition of VEGF expression and decrease of invasion markers such as MMP-2, MMP-9, and collagen, leading to an increase of survival. Our results showed significant decrease of angiostatin and MMP-9 expression during 21 days of treatment when the tumor size was smallest in size. These results are in accordance with several investigators suggesting a dual effect of MMPs synthesized by EC. MMP-9 secretion can facilitate extracellular matrix degradation and new blood vessel formation, on the other hand, blocking of angiogenesis by producing inhibitors of EC growth, such as angiostatin, promoting auto-inhibition of angiogenesis [34]–[37]. However, 28 days after treatment there was an increase in angiostatin followed by increase of the tumor size and decreased in MMP-9 level, indicating development of resistance to the treatment and suggesting that angiostatin might have acted in a compensatory role.

## Conclusion

The angiogenic response involves many kind of factors that promotes cell proliferation and modulate others processes as cell migration, matrix degradation, recruitment and differentiation of EC and their precursors. Similar way, our study showed that HET0016 treatment is effective to inhibit tumor growth on early and delayed treatment for 21 days, following a decrease protein expression of several pro-angiogenic factors. However, when the delayed treatment group is followed for 28 days, the tumor seems to acquire resistance to treatment, leading to tumor growth and increased expression of factors such as angiogenin, angiostatin, angiopoietin-1/2, EGF-R and IGF-1.

## Supporting Information

S1 Fig **Toxicity study for HET0016 in cancerous and non-cancerous breast cells.** HET0016 has significant toxicity only on cancerous cells.
(DOCX)
