# Supplementary material for: HET0016, a Selective Inhibitor of 20-HETE Synthesis, Decreases Pro-Angiogenic Factors and Inhibits Growth of Triple Negative Breast Cancer in Mice
Source: PLoS One. 2014 Dec 30;9(12):e116247. doi: 10.1371/journal.pone.0116247 (PMC4280215; doi:10.1371/journal.pone.0116247)
Supplement: S1 Fig — Toxicity study for HET0016 in cancerous and non-cancerous breast cells. HET0016 has significant toxicity only on cancerous cells. (DOCX) [file pone.0116247.s001.docx]

Hundred thousand triple negative breast cancer (MDA-MB-231) and non-tumorigenic breast (MCF-10A) cells per mL were subjected to different doses of HET0016 (5µM and 100µM) for four hours in serum free media. Cell viability was determined by trypan blue dye exclusion tests. The experiments were repeated by two independent investigators on two different dates. HET0016 significantly decreased cell viability only on MDA-MB-231 cells. No effect was observed on MCF-10A cells. Please see the Supplement Figure 1.


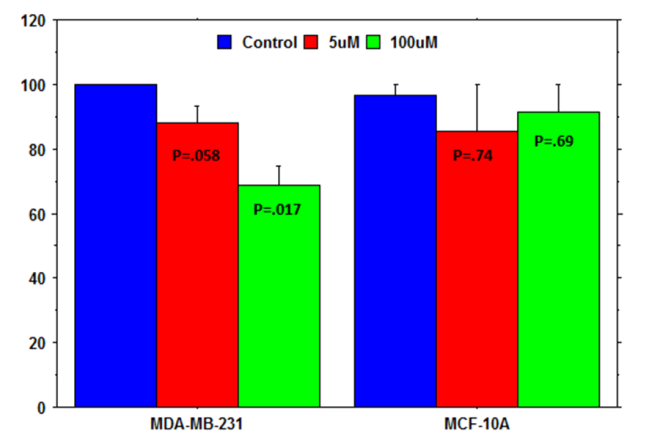


**Figure S1:** **Toxicity study for HET0016 in cancerous and non-cancerous breast cells**. HET0016 has significant toxicity only on cancerous cells.
